# Diagnostic and prognostic value of serum soluble suppression of tumorigenicity-2 in heart failure with preserved ejection fraction: A systematic review and meta-analysis

**DOI:** 10.3389/fcvm.2022.937291

**Published:** 2022-09-20

**Authors:** Yujiao Shi, Jiangang Liu, Chunqiu Liu, Xiong Shuang, Chenguang Yang, Wenbo Qiao, Guoju Dong

**Affiliations:** ^1^Department of Post-graduate Institute, Chinese Academy of Traditional Chinese Medicine, Beijing, China; ^2^National Clinical Research Center for Chinese Medicine Cardiology, Xiyuan Hospital, Chinese Academy of Traditional Chinese Medicine, Beijing, China; ^3^Department of Cardiovascular Internal Medicine, Xiyuan Hospital, Chinese Academy of Traditional Chinese Medicine, Beijing, China

**Keywords:** soluble suppression of tumorigenicity 2, diastolic heart failure, heart failure with preserved ejection fraction, diagnosis, prognosis, meta-analysis

## Abstract

**Background:**

Heart failure (HF) with preserved ejection fraction (HFpEF) is a growing public health burden, with mortality and rehospitalization rates comparable to HF with reduced ejection fraction (HFrEF). The evidence for the clinical usefulness of soluble suppression of tumorigenicity 2 (sST2) in HFpEF is contradictory. Therefore, we conducted the following systematic review and meta-analysis to assess the diagnostic and prognostic value of serum sST2 in HFpEF.

**Methods:**

PubMed and Scopus were searched exhaustively from their inception until March 15, 2022. In diagnostic analysis, we compared the diagnostic value of serum sST2 in HFpEF to NT pro-BNP. We separately pooled the unadjusted and multivariate-adjusted hazard ratios (HRs) and the corresponding 95% confidence intervals (CIs) in prognostic analysis.

**Results:**

A total of 16 publications from 2008 to 2021 were examined. The results of this analysis were as follow: Firstly, compared with NT pro-BNP, sST2 obtains poor diagnostic performance in independently identifying HFpEF from healthy controls, hypertensive patients, and HFrEF patient. Nevertheless, it may provide incremental value to other biomarkers for diagnosing HFpEF and deserves further investigation. Secondly, log sST2 was independently associated with adverse endpoints on multivariable analysis after adjusting for variables such as age, sex, race, and NYHA class. Per log unit rise in sST2, there was a 2.76-fold increased risk of all-cause death [HR:2.76; 95% CI (1.24, 6.16); *p* = 0.516, *I*^2^ = 0%; *P* = 0.013] and a 6.52-fold increased risk in the composite endpoint of all-cause death and HF hospitalization [HR:6.52; 95% CI (2.34, 18.19); *p* = 0.985, *I*^2^ = 0%; *P* = 0.000]. Finally, the optimal threshold levels of serum sST2 need further determined.

**Conclusions:**

Higher sST2 was strongly linked to an increased risk of adverse outcomes in HFpEE. Especially, log sST2 independently predicted all-cause death and the composite endpoint of all-cause death and HF hospitalization. However, prospective and multicenter studies with large-sample and extended follow-up periods are required to validate our results due to limitations in our research.

## Introduction

Heart failure (HF), a complex and heterogeneous medical syndrome characterized by structural and functional cardiac abnormalities and hemodynamic disruptions, represents the end-stage manifestation of numerous cardiovascular disorders (1). HF is categorized into three groups based on the measurement of the left ventricular (LV) ejection fraction (LVEF) according to the European Society of Cardiology (ESC) Guidelines issued in 2021: HF with reduced ejection fraction (HFrEF, LVEF ≤ 40%), HF with mildly reduced ejection fraction (HFmrEF, LVEF 41–49%), and HF with preserved ejection fraction (HFpEF, LVEF ≥ 50%) (2). HFpEF, which affects approximately half of all HF patients worldwide, is increasing in prevalence and is associated with an elevated risk of hospitalization and mortality (3). The pathogenic mechanism of HFpEF remains poorly understood, which makes it difficult to establish a precise clinical diagnosis and choose an appropriate treatment (4–6). As a result, early and accurate diagnosis of HFpEF and determining the prognosis of HFpEF patients can contribute to adopting appropriate interventions to slow or halt disease progression.

Circulating biomarkers reflect the pathophysiological processes involved in the occurrence and development of HF: myocardial insult, inflammation, necrosis, fibrosis, and ventricular reconstruction, and thus play a pivotal role in diagnosing HF, severity stratification, monitoring treatment response, and evaluation of prognosis (7). N-terminal pro-B-type natriuretic peptide (NT-proBNP) released by cardiac muscle tissue in response to abnormal volume load is an established indicator for the diagnosis and prognosis of HFrEF. Unfortunately, NT-proBNP elevation is not universal in HFpEF, thus limiting its usefulness in HFpEF (8, 9). Soluble suppression of tumorigenicity-2 (sST2) is thought to be implicated in inflammation, cardiomyocyte hypertrophy or apoptosis, and myocardial interstitial fibrosis (10). Serum sST2 is emerging as a potentially valuable biomarker, providing additional diagnostic and prognostic value in HF (11). However, the existing clinical research exploring the diagnostic and prognostic role of serum sST2 in HFpEF is limited, and its results are contradictory. We, therefore, performed a systematic review and meta-analysis to evaluate the diagnostic and prognostic significance of serum sST2 in HFpEF.

## Materials and methods

### Literature search strategy

A systematic review and meta-analysis was conducted according to PRISMA (Preferred Reporting Items for Systematic Reviews and Meta-Analysis) guidelines published in 2020 (12). Two researchers (Shi and Liu) conducted a comprehensive literature search using two electronic databases (PubMed and Scopus). We searched for studies in English published from the inception of each database until March 15, 2022. The terms “Heart Failure, Diastolic,” “Heart Failure with Preserved Ejection Fraction,” “Diastolic Dysfunction,” “Preserved Ejection Fraction,” “Biomarkers,” “Soluble Suppression of Tumorigenicity 2,” “sST2,” “ST2,” and “Soluble ST2” were utilized based on the rule of each database. For PubMed, the following search was performed: ((((Heart Failure, Diastolic [MeSH Terms]) OR (Heart Failure with Preserved Ejection Fraction [Title/Abstract])) OR (Diastolic Dysfunction [Title/Abstract])) OR (Preserved Ejection Fraction [Title/Abstract])) AND (((((Biomarkers [Title/Abstract]) OR (Soluble Suppression of Tumorigenicity 2 [Title/Abstract])) OR (sST2 [Title/Abstract])) OR (ST2 [Title/Abstract])) OR (Soluble ST2 [Title/Abstract])).

### Literature inclusion and exclusion criteria

The inclusion criteria for this study were as follows: (i) diagnostic criteria: Meeting the diagnostic criteria for HF, patients with HFpEF had an LVEF ≥ 50%, while HFrEF had an LVEF ≤ 40% (2); (ii) study design: prospective and retrospective observational studies (cohort studies, case-control studies, and cross-sectional studies); (ii) endpoints: diagnostic values of serum sST2 in distinguishing HFpEF from controls (healthy controls, hypertensive patients, and HFrEF patients) and association of serum sST2 with adverse endpoints in HFpEF patients [all-cause death and the composite endpoint of all-cause death and HF hospitalization or cardiovascular (CV) death and HF hospitalization]. The exclusion criteria for this study were as follows: (i) irrelevant or duplicated studies; (ii) the papers were case reports, reviews, letters, conference abstracts, commentaries, editorials, or non-human studies; (iii) the articles lacked full text or sufficient raw data.

### Literature quality evaluation and data extraction

Two independent reviewers (Yang and Qiao) assessed the quality of the included studies using the Newcastle–Ottawa Quality Assessment Scale (NOS) system, a “star-based” grading system comprised of three parts (selection, comparability, and outcomes). The total NOS score ranged from 0 to 9, with research scoring six or above considered high quality.

Two separate researchers (Shi and Xiong) extracted relevant data from the included studies and entered them into specifically constructed Microsoft Excel spreadsheets. The extracted contents were as follows: (i) information on the publication: the last name of the first author, the year of publication, and the country setting; (ii) demographic characteristics: sample size, males proportions, mean age, and mean (standard deviation, *SD*) or median (interquartile range, IQR) values of LVEF; (iii) study details: study design, serum sST2-related data (assay kits, measurement methods, and units), data on the diagnostic analysis [definition of the control group, sample size, comparison of diagnostic value of sST2 and NT-proBNP [mean (*SD*) or median (IQR) values, the optimal cut-off value, area under the curve (AUC) for the receiver operating characteristic curve (ROC), sensitivity, and specificity], and data regarding the prognostic meta-analysis [follow-up duration, clinical outcome, unadjusted and multivariable-adjusted hazard ratios (HRs), 95% confidence intervals (CIs), and adjustment variables]; (iv) NOS quality scores. Disagreements were resolved by mutual coordination or third-party adjudication (Dong and Liu).

### Statistical analysis

STATA (Version 16.0) was used to assess the association between serum sST2 and unfavorable endpoints in HFpEF patients, with combined HRs and 95% CIs representing the effect sizes. We separately pooled the unadjusted and multivariate-adjusted HRs and the corresponding 95% CIs. The heterogeneity was examined by the Cochran Q statistics (*P* < 0.1 was considered statistical heterogeneity) and *I*^2^ Statistics (25, 50, and 75% were considered to represent low, medium, and high heterogeneity, respectively). When the Q test (*I*^2^ ≥ 50% or *p* < 0.05) demonstrated significant heterogeneity across trials, a random-effect model was utilized; otherwise, the fixed-effects model was used. If considerable heterogeneity (*I*^2^ ≥ 50%) was identified among included studies, subgroup analyses were performed to explore possible sources of heterogeneity. Subgroup analyses were performed based on sST2 value change (per log unit increase or per unit increase), Ethnicity (Asian or Western), sex (50% male), study design (single or multicenter study), serum sST2 detection method (ELISA or multiplexed assay), sST2 unit (ng/ml or pg/ml), and length of follow-up (24 months). Publication bias was evaluated using the Funnel plot and Egger's test. A sensitivity analysis was employed to estimate the influence of a single study on the total estimate by eliminating one study at a time. A *p*-value of < 0.05 was considered statistically significant.

## Results

### Literature search results

**Figure 1:**
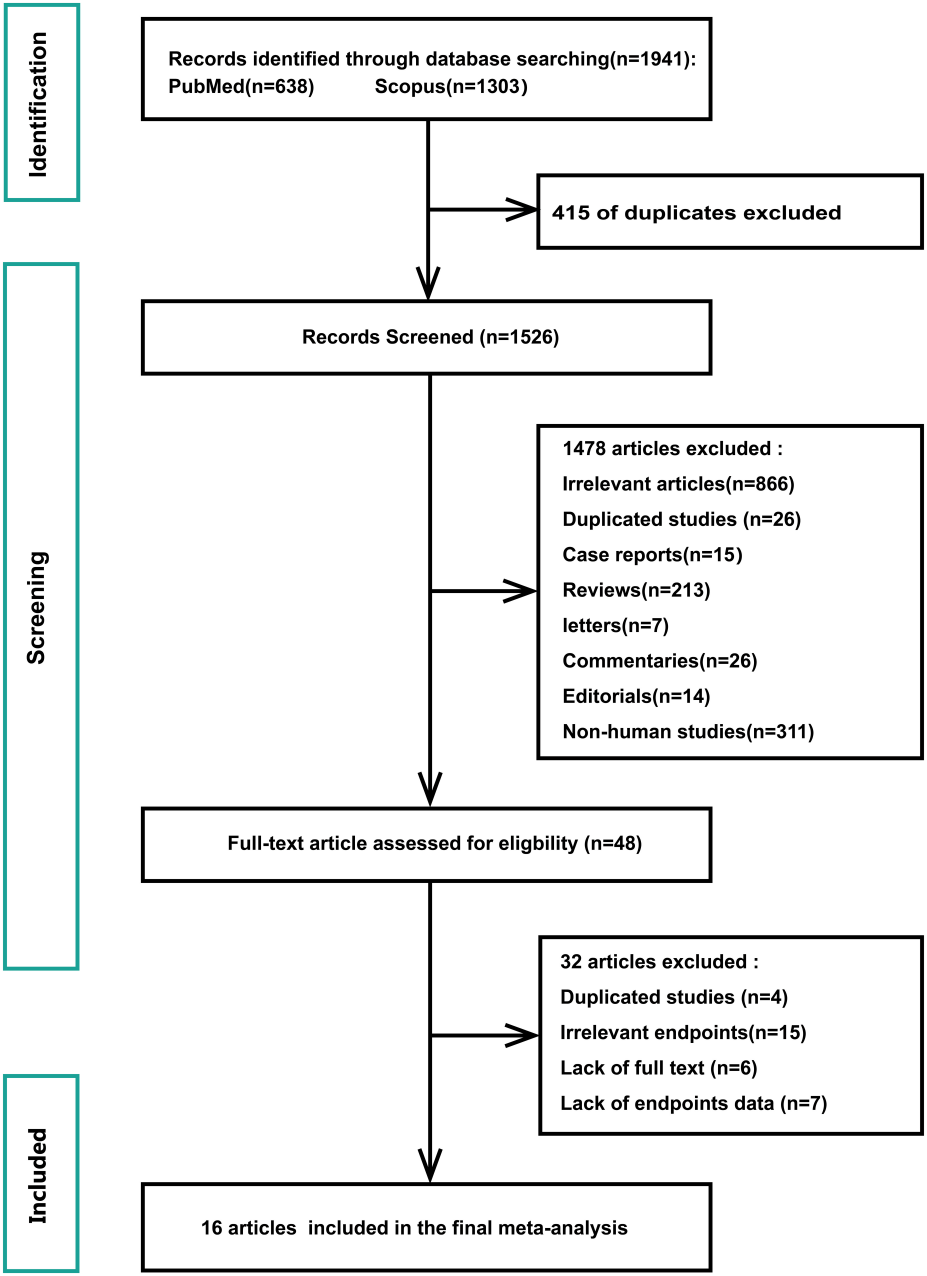
Flowchart of database search and text screening procedure.

Figure 1 shows a flowchart of the database search and text screening procedures. A total of 1,941 publications (638 from PubMed and 1,303 from Scopus) were retrieved through database searching. We reviewed the titles and abstracts of 1,526 articles after eliminating 415 duplicates. Following the inclusion and exclusion criteria, 1,478 articles were then deleted. Finally, two independent researchers (Shi and Liu) read the full text of the remaining 48 papers and excluded 32 records owing to redundant research, irrelevant findings, and inadequate data. The meta-analysis included a total of 16 publications.

### Characteristics of included studies

Table 1 shows the baseline characteristics of the selected research. Sixteen publications from 2008 to 2021 were examined, comprising 14 prospective cohort studies and two prospective cross-sectional studies. Six of those were multicenter studies, while the remaining ten were single-center studies. A total of 2,761 patients (including 2,483 HFpEF patients and 278 control groups) were involved, of whom 1,349 were males, with an average age of 70.02 years. The included studies used various sources of sST2 reagents and adopted diverse detection strategies (e.g., ten studies used ELISA to detect sST2, three used sandwich ELISA, and the remaining three used Luminex® bead-based multiplex assays). Besides the above, the dose units varied in different studies, ultimately culminating in substantial differences in serum sST2 values. Concerning the purpose of the study, five studies explored the diagnostic value of serum sST2 in distinguishing HFpEF from controls, while twelve assessed its association with poor endpoints in HFpEF patients. The included studies had NOS values ranging from 6 to 9, indicating that the methodological quality was credible (Supplementary Table 1).

**Table 1:** Baseline characteristics of the 16 selected research.

**References**	**Country**	**Sample size, *n***	**HFpEF patients, *n***	**Males, *n***	**Age, mean, years**	**LVEF of HFpEF patients, mean (SD)/median (IQR), %**	**Study design**	**Source of sST2 assay kit**	**sST2 detection method and unit**	**sST2 of HFpEF patients mean (SD)/media*n* (IQR), %**	**Purpose**	**NOS Scores**
Cui et al. (13)	China	247	172	112	70.33	60 (56, 62)	Prospective cohort, single-center	Qiyi Biological Co, Shanghai, China	ELISA, pg/ml	63.48 (49.55, 86.54)	Diagnosis, prognosis	8
Santhanakrishnan et al. (14)	Singapore	151	50	96	63.67	60 ± 7	Prospective, cross-sectional, single-center	Critical Diagnostics, San Diego, CA, USA	Sandwich ELISA, ng/mL	31.52 (24.55, 51.95)	Diagnosis	8
Wang et al. (15)	Taiwan.	107	68	57	64	68 ± 7	Prospective, cross-sectional, single-center	R&D Systems, Minneapolis, Minnesota	ELISA, ng/mL	17.9 ± 67.9	Diagnosis	7
Pan et al. (16)	China	85	60	44	68.14	57 ± 5	Prospective cohort, single-center	Wuhan Boshide Biological Company	ELISA, ng/mL	1.31 (0.30, 2.80)	Diagnosis	7
Sinning et al. (17)	Germany	108	70	65	65.5	64 (59, 70)	Prospective cohort, single-center	Critical Diagnostics	Sandwich ELISA, ng/mL	26.5 (21.7, 36.0)	Diagnosis	8
Najjar et al. (18)	Sweden	86	86	42	73	64 (58, 68)	Prospective cohort, multicenter	Assay kit; Critical Diagnostics, CA, USA	ELISA, ug/L	23 (17, 31)	Prognosis	7
Shah et al. (19)	US	200	200	50	55	—	Prospective cohort, multicenter	Critical Diagnostics	ELISA, U/mL	31.7 (23.7–55.7)	Prognosis	8
Gao et al. (20)	China	380	380	188	71	59 (53, 65)	Prospective cohort, single-center	R&D Systems, Minneapolis, MN, USA	Luminex Bead-Based multiplex assay, ng/mL	—	Prognosis	8
Manzano-Fernández et al. (21)	Spain	197	197	83	74	60 (55, 65)	Prospective cohort, multicenter	—	ELISA, ng/mL	0.38 (0.26, 0.79)	Prognosis	7
Sugano et al. (22)	Japan	191	191	99	76.1	60.0 ± 7.6	Prospective cohort, multicenter	R&D Systems, Minneapolis, MN, USA	ELISA, pg/ml	18.0 (11.9, 26.2)	Prognosis	6
Roy et al. (23)	Belgium	143	143	87	78	63 ± 7	Prospective cohort, Single-center	Critical Diagnostics, CA, USA	ELISA, ng/mL	42 (31, 60)	Prognosis	7
Song et al. (24)	China	110	110	63	69.4	61 ± 6	Prospective cohort, Single-center	Critical Diagnostics, California, USA.	ELISA, ng/mL	40.5 (22.0–63.7)	Prognosis	6
Sanders-van Wijk et al. (25)	Switzerland	112	112	42	80	57 ± 6	Prospective cohort, Multicenter	—	ELISA, ng/mL	37.6 (28.5–54.7)	Prognosis	9
Chirinos et al. (26)	USA	379	379	203	70	—	Prospective cohort, Multicenter	Bristol-Myers-Squibb; Ewing Township, NJ	Luminex® Bead-Based multiplexed assay, pg/mL	—	Prognosis	8
Kanagala et al. (27)	US	130	130	65	72.5	56 ± 6	Prospective cohort, Single-center	—	Luminex® bead-based multiplex assay, ng/mL	—	Prognosis	8
Moliner et al. (28)	Spain	135	135	53	69.6	60 ± 8	Prospective cohort, Single-center	Critical Diagnostics, San Diego, CA, USA	Sandwich ELISA, ng/mL	44.4 (32.3–57.3)	Prognosis	8

HFpEF, Heart failure with preserved ejection fraction; LVEF, left ventricular ejection fraction; sST2, soluble suppression of tumorigenicity-2.

### Diagnostic value of serums SST2 in identifying HFpEF from controls compared with NT pro-BNP

As shown in Tables 2, 3, five studies (13–17) estimated the diagnostic value of serum sST2 in identifying HFpEF from controls [healthy controls (2 studies), hypertensive patients (1 study), and HFrEF (4 studies)] compared to NT pro-BNP. 420 HFpEF patients, 80 controls, 39 hypertensive patients, and 159 HFrEF patients were enrolled. Cui et al. and Santhanakrishnan et al. demonstrated that sST2 performed worse than NT pro-BNP at distinguishing HFpEF from healthy controls, with an AUC for ROC of < 0.7 and lower sensitivity and specificity (13, 14). Although Wang et al. found that sST2 (AUC 0.80) performed more successfully in identifying HFpEF from hypertensive patients than NT pro-BNP (AUC 0.70), the area under the ROC curve comparisons did not display statistical significance (*P* = 0.301) (15). Regarding the differentiation of HFpEF from HFrEF (13, 14, 16, 17), the overall diagnostic performance of NT-proBNP was significantly superior to that of sST2, with an AUC as high as 0.901 (sensitivity: 60.5%; specificity: 80%) when the optimal threshold value was 295.85 pg/ml (13). These findings indicate that, compared to NT pro-BNP, sST2 showed poor performance in independently identifying HFpEF from healthy controls, hypertensive patients, and HFrEF patients.

**Table 2:** Baseline characteristics of the 5 studies for diagnostic analysis.

**References**	**HFpEF patients**				**Controls**				**HFrEF patients**			
	**Sample size, *n***	**LVEF, mean (SD)/median (IQR), %**	**sST2, mean (SD)/median (IQR)**	**NT-proBNP, mean (SD)/median (IQR), pg/ml**	**Sample size, *n***	**Definition of the control group**	**sST2, mean (SD)/median (IQR)**	**NT-proBNP, mean (SD)/median (IQR), pg/ml**	**Sample size, *n***	**LVEF, mean (SD)/median (IQR), %**	**sST2, mean (SD)/median (IQR)**	**NT-proBNP, mean (SD)/median (IQR), pg/ml**
Cui et al. (13)	172	60 (56, 62)	63.48 (49.55, 6.54) pg/ml	614 (242.5, 478.5)	30	Healthy controls from the physical examination center	61.7 (50, 70) pg/ml	189 (132.5, 213.75)	45	31 (28,35)	140.2 (81.14, 164.7) pg/ml	330 (1746.5, 10,013)
Santhanakrishnan et al. (14)	50	66 + 7	31.52 (24.55, 51.95) ng/ml	942 (309, 2,768)	50	Healthy controls with age ≥55 years	27.58 (21.50, 32.79) ng/ml	69 (41, 102)	51	25 ± 10	35.25 (28.14, 53.62) ng/ml	2,562 (1,038, 6,373)
Wang et al. (15)	68	68 + 7	17.9 ± 6 7.9 ng/mL	71 ± 53	39	Hypertensive patients	17.9 ± 7.9 ng/mL	262 ± 470				
Pan et al. (16)	60	57 ± 5	1.31 (0.30, 2.80) ng/ml	2346.50 (838.77, 8164.00)					25	33 ± 4	5.26 (2.82, 7.56) ng/ml	5934.00 (2871.50, 15520.50)
Sinning et al. (17)	70	64 (59, 70)	26.5 (21.7, 36.0) ng/ml	145.5 (75.5, 293.9)					38	43 (36, 48)	29.6 (23.4, 43.3) ng/ml	955.7 (243.6, 1876.7)

**Table 3:** Diagnostic value of serums sST2 in identifying HFpEF from controls compared with NT pro-BNP.

**Study**	**sST2**					**NT-proBNP**				
	**Cut-off**	**AUC (95%CI)**	**Sensitivity, %**	**Specificity, %**	***P-value***	**Cut-off**	**AUC (95%CI)**	**Sensitivity, %**	**Specificity, %**	***P-value***
**HFpEF vs. controls**										
Cui et al. (13)	68.6 pg/ml	0.584 (0.49, 0.68)	48	57	0.17	295.85 pg/ml	0.806 (0.66, 0.82)	60.5	80	0.000
Santhanakrishnan et al. (14)	26.47 ng/ml	0.662 (0.55–0.77)	78	45	0.005	247.60 pg/ml	0.934 (0.886–0.983)	82	94	<0.001
Wang et al. (15)	13.5 ng/ml	0.80 (0.7–0.89)	74	74	<0.001	—	0.70 (0.58, 0.79)	—	—	0.003
**HFpEF vs. HFrEF**										
Cui et al. (13)	68.6 pg/ml	0.824 (0.73, 0.90)	82	56	0.000	295.85 pg/ml	0.901 (0.85, 0.96)	95	60	0.000
Santhanakrishnan et al. (14)	23.18 ng/ml	0.624 (0.514–0.733)	69	50	0.379	247.60 pg/ml	0.689 (0.586–0.792)	67	70	0.001
Pan et al. (16)	0.332 ng/ml	0.717 (0.628–0.796)	51.7	95	<0.01	799.750 pg/ml	0.881 (0.809–0.933)	78.3	96.7	<0.01
Sinning et al. (17)	—	0.586	—	—	—	—	0.737	—	—	—

### Association of serums SST2 with adverse outcomes in HFpEF patients

**Figure 2:**
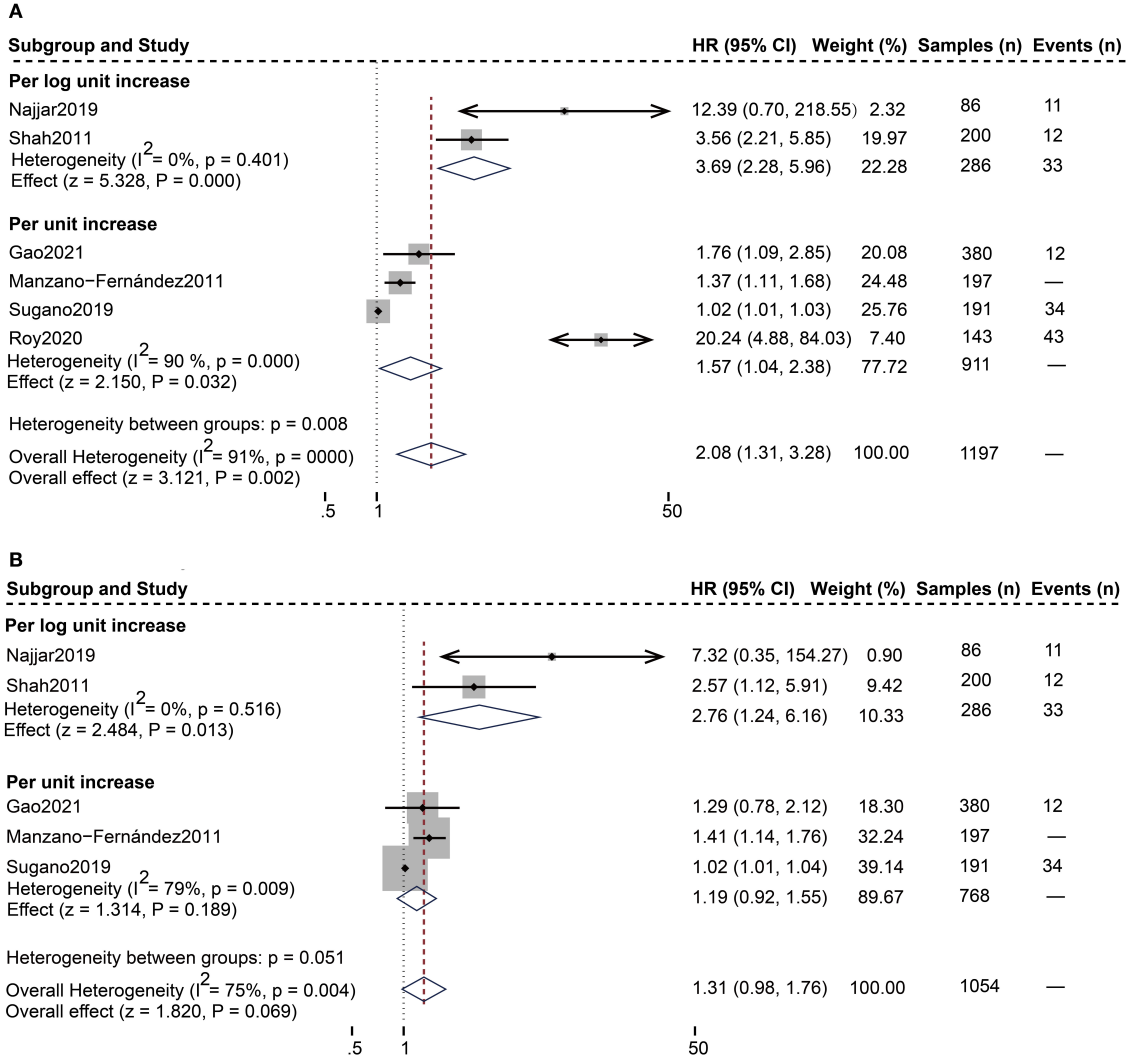
Forest plot of the association between serum sST2 and all-cause death. **(A)** Univariate analysis in the prediction of all-cause death. **(B)** Multivariate analysis in the prediction of all-cause death.

As shown in Table 4, 12 studies (13, 18–28) assessed the correlation between serum sST2 and adverse endpoints. During a mean follow-up period of 12–79.2 months, all-cause death was known to occur in 112 of 2,235 HFpEF patients, while 328 patients experienced the composite endpoint of all-cause death and HF hospitalization. In unadjusted analysis, Higher serum sST2 was strongly associated with an increased risk of all-cause mortality [Random-effects model, HR 2.08; 95% CI (1.31, 3.28); *p* = 0.000, *I*^2^ = 91%; *P* = 0.002]. Following subgroup analysis depending on changes in sST2 values, both per log unit rise [HR 3.69; 95% CI (2.28, 5.96); *p* = 0.401, *I*^2^ = 0%; *P* = 0.000] and per unit rise [HR 1.57; 95% CI (1.04, 2.38); *p* = 0.000, *I*^2^ = 90%; *P* = 0.032] were related to increased risk. Further subgroup analysis of revealed that sST2 unit (ng/ml) and follow-up time >24 months were a source of heterogeneity and associated with a high risk of death (Supplementary Table 2). In multivariate-adjusted analysis, we only found that per log unit rise in sST2 is related to a 2.76-fold increased risk of all-cause death [HR:2.76; 95% CI (1.24, 6.16); *p* = 0.516, *I*^2^ = 0%; *P* = 0.013] (Figure 2).

**Table 4:** Univariate and multivariate analysis in the prediction of all-cause death and the composite endpoints.

**References**	**HFpEF patients**	**Follow up duration (months)**	**Clinical outcome**	**Univariable analysis**		**Multivariable analysis**		**Variables adjusted**
				**HR (95% CI)**	***P***	**HR (95% CI)**	***p***	
**All-cause death**								
**sST2 as continuous variables**								
Najjar et al. (18) per log unit increase	86	17.4	11 all-cause death	12.39 (0.70–218.55)	0.086	7.32 (0.35–154.27)	0.2	Age, sex, and NYHA class.
Shah et al. (19) per log unit increase	200	12	20 all-cause death	3.56 (2.21–5.85)	0.001	2.57 (1.12–5.91)	0.03	Age, sex, BMI, systolic blood pressure, diastolic blood pressure, heart rate, eGFR, history of (hypertension, CAD, T2DM, AF), prescription of (ACEI, β-blockers, digoxin, diuretic, bronchodilator), CRP, and NT-proBNP.
Gao et al. (20) per unit increase	380	24	102 all-cause death	1.76 (1.09–2.85)	0.021	1.29 (0.78–2.12)	0.325	Age, sex, race, smoking status, systolic blood pressure, heart rate, left ventricular hypertrophy, history of CAD, serum glucose, creatinine, albumin levels, and NT-pro BNP.
Manzano-Fernández et al. (21) per unit increase	197	12	All-cause death	1.37 (1.11–1.68)	0.003	1.41 (1.14–1.76)	0.002	Age, BMI, systolic or diastolic blood pressure, LVEF, NYHA class, history of heart failure, prescription of β-blockers or ACEI, hemoglobin, leukocytes, eGFR, blood urea nitrogen, CRP, and NT-pro BNP.
Sugano et al. (22) per unit increase	191	14.83	34 all-cause death	1.02 (1.01–1.03)	<0.001	1.02 (1.009–1.04)	0.002	Age and sex.
Roy et al. (23) per unit increase	143	30	43 all-cause death	20.24 (4.88–84.03)	<0.001			
**sST2 as dichotomous variables**								
Manzano-Fernández et al. (21) (>0.35 ng/ml)	197	12	All-cause death			3.26 (1.50–7.05)	0.003	Described above.
Manzano-Fernández et al. (21) (0.33–0.71) ng/ml	197	12	51 all-cause death	2.67 (1.6–6.15)	<0.001	2.63 (1.13–6.12)	<0.001	Described above.
Manzano-Fernández et al. (21) ≥0.72 ng/ml	197	12	69 all-cause death	4.07 (1.77–9.35)	<0.001	4.18 (1.79–9.35)	<0.001	Described above.
**All-cause death or HF hospitalization**								
**sST2 as continuous variables**								
Najjar et al. (18) per log unit increase	86	17.4	36 all-cause death or HF hospitalization	10.04 (1.89–53.44)	0.007	6.62 (1.04–42.28)	0.046	Described above.
Song et al. (24) per log unit increase	110	12	13 all-cause death and 19 HF hospitalization	7.07 (2.30–21.72)	0.001	6.48 (1.89–22.21)	0.003	Age, sex, smoking status, systolic blood pressure, NYHA class, and history of (T2DM and CAD).
Sanders-van Wijk et al. (25) per log unit increase	112	18	39 all-cause death and HF hospitalization	12.18 (2.45–60.65)	0.002			
Roy et al. (23) per unit increase	143	30	87 all-cause death and HF hospitalization	3.46 (1.23–9.74)	0.020			
Chirinos et al. (26) per unit increase	379	34.32	94 all-cause death and HF hospitalization	1.42 (1.15–1.75)	0.001	1.32 (1.06–1.64)	0.0117	Age, sex, BMI, smoking status, LVEF, NYHA class, history of (T2DM, chronic obstructive pulmonary disease, heart failure duration>18 months), prescription of β-blockers or ACEI, and creatinine.
Kanagala et al. (27) per unit increase	130	47.6	21 all-cause death and 40 HF hospitalization	1.275 (0.990–1.641)	0.060			
Moliner et al. (28) per unit increase	135	79.2	All-cause death and HF hospitalization	1.11 (0.86–1.43)	0.44			
**sST2 as dichotomous variables**								
Song et al. (24) 63.7 ng/mL	439	12	57 all-cause death and 82 HF hospitalization	4.08 (1.52–10.96)	0.005	3.73 (1.36–10.26)	0.011	Described above.
**CV death or HF hospitalization**								
Cui et al. (13) per log unit increase	172	12	CV death and HF hospitalization			1.34 (1.14, 1.57)	0.089	Age, sex, systolic blood pressure, diastolic blood pressure, heart function of grade NYHA, left ventricular ejection fraction, coronary artery disease, hypertension, β-blockers treatment, aldosterone receptor antagonist, LDL, and eGFR.
Moliner et al. (28) per log unit increase	135	79.2	CV death and HF hospitalization	1.04 (0.80–1.35)	0.79			

HFpEF, heart failure with preserved ejection fraction; HR, hazard ratios; CI, confidence intervals; CV, cardiovascular; NYHA, New York Heart Association; BMI, body Mass Index; eGFR, glomerular filtration rate; CAD, coronary artery disease; T2DM, type 2 diabetes mellitus; AF, atrial fibrillation; ACEI, angiotensin-converting enzyme inhibitors; CRP, C-reactive protein; NT-proBNP, N-terminal pro-B-type natriuretic peptide; LVEF, left ventricular ejection fraction; LDL, low-density lipoprotein.

**Figure 3:**
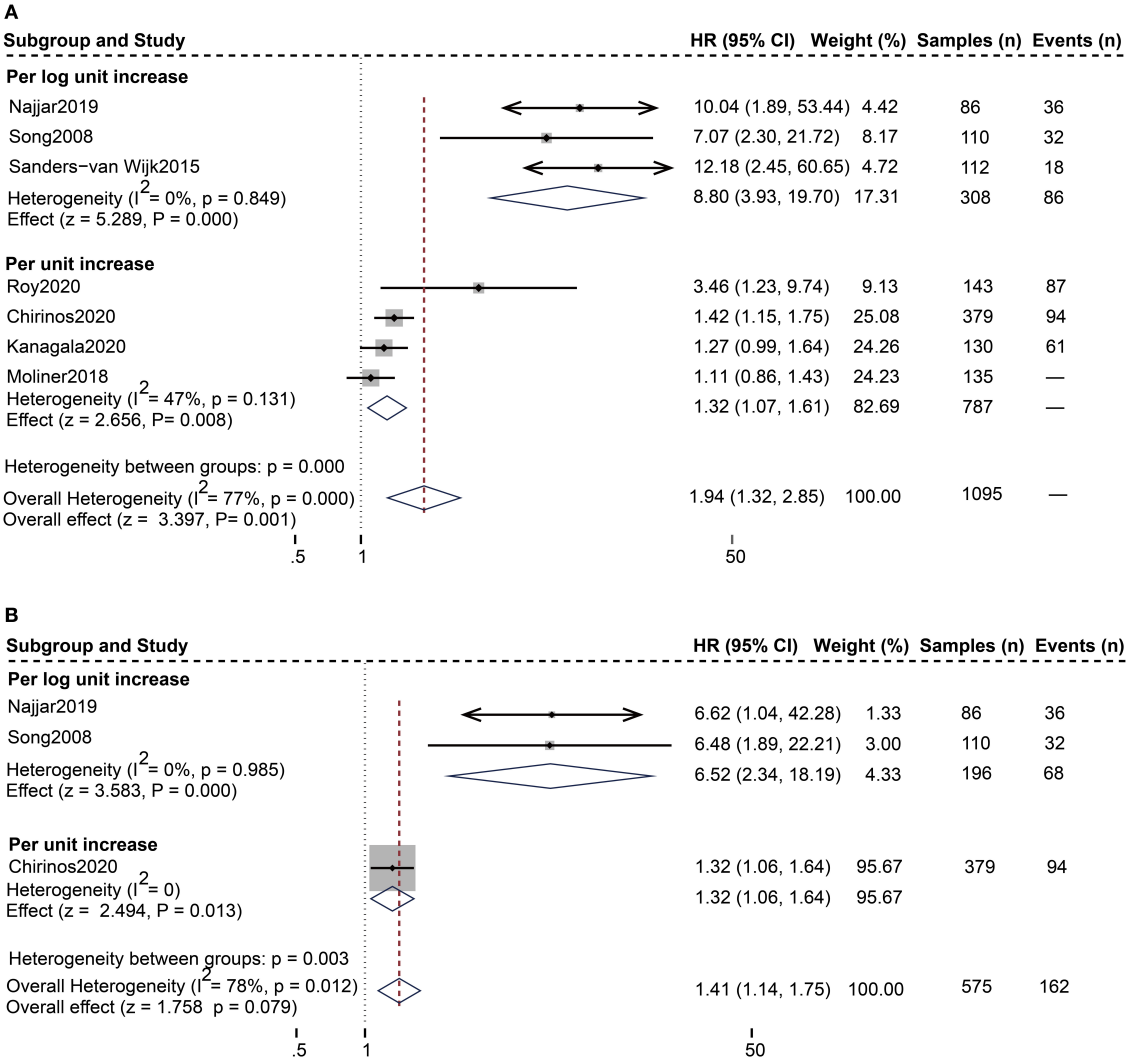
Forest plot of the association between serum sST2 and the composite outcome of all-cause death and HF hospitalization. **(A)** Univariate analysis in the prediction of the composite outcome of all-cause death and HF hospitalization. **(B)** Multivariate analysis in the prediction of the composite outcome of all-cause death and HF hospitalization.

For the composite endpoint, on univariate assessment, higher serum sST2 was substantially related to the composite endpoint of all-cause death and HF hospitalization [Random-effects model, HR:1.94; 95% CI (1.32, 2.85); *p* = 0.000, *I*^2^ = 77%; *P* = 0.001]. After subgroup analysis based on changes in sST2 values, both per log unit rise [HR 8.80; 95% CI (3.93, 19.70); *p* = 0.849, *I*^2^ = 0%; *P* = 0.000] and per unit rise [HR 1.32; 95% CI (1.07, 1.61); *p* = 0.131, *I*^2^ = 47%; *P* = 0.008] were related to increased risk. on multivariate-adjusted assessment, we only confirmed that per log unit rise in sST2 is associated with a 6.52-fold increased risk of the composite endpoint of all-cause death and HF hospitalization [HR:6.52; 95% CI (2.34, 18.19); *p* = 0.985, *I*^2^ = 0%; *P* = 0.000] (Figure 3). Conversely, according to studies by Cui et al. and Moliner et al. neither unadjusted nor multivariate-adjusted analyses discovered a correlation between sST2 and the composite outcome of CV death and HF hospitalization (13, 28).

The assessment of publication bias regarding all-cause death and the composite endpoint of all-cause death and HF hospitalization showed that the Funnel plots were asymmetric (Supplementary Figures 1A, 2A), and the *p*-values of the Egger's test were < 0.05 (*p* = 0.007 and 0.003, respectively) (Supplementary Figures 1B, 2B), suggesting notable publication bias. The sensitivity analysis indicated that none of the studies significantly affected the pooled estimates (Supplementary Figures 1C, 2C).

## Discussion

This systematic review and meta-analysis revealed that although serum sST2 obtains poor diagnostic performance in independently discriminating HFpEF patients from healthy people, hypertensive patients, and HFrEF, higher serum sST2 values were significantly related to an increased risk of adverse endpoints in HFpEF patients. Log sST2 was independently associated with adverse endpoints after adjusting for variables such as age, sex, race, and NYHA class on multivariable analysis. Per log unit rise in sST2, there was a 2.76-fold increased risk of all-cause death and a 6.52-fold increased risk in the composite endpoint of all-cause death and HF hospitalization.

With an increasing prevalence of comorbidities such as obesity, hypertension, coronary artery disease, diabetes mellitus, chronic kidney disease, and chronic obstructive pulmonary disease, HFpEF is becoming a significant challenge for clinicians (29–31). HFpEF is a frequent cause of hospital admissions for persons aged 65 or older, leading to substantial mortality and high medical expenses (32, 33). Currently, the diagnosis of HFpEF depends on the presence of symptoms and/or signs of HF, LVEF ≥ 50%, and objective evidence of structural and/or functional cardiac defects (e.g., LV remodeling, increased LV filling pressures, and LV diastolic dysfunction) (34). Standard diagnostic algorithms primarily rely on echocardiography (E/e' ratio and pulmonary capillary wedge pressure) and biomarker assessment (NT-proBNP) (35). However, when tested prospectively, this assessment technique provided excellent specificity but low sensitivity (36, 37), resulting in only a small number of patients with HFpEF being identified, and those with HFpEF in the initial stages are easily ignored. Furthermore, the diagnostic and prognostic value of NT-proBNP in HFrEF has been well-established (38), while it is controversial in HFpEF (27, 28). Therefore, it is essential to explore effective biomarkers for early diagnosis, prognosis assessment, and treatment monitoring in patients with HFpEF.

**Figure 4:**
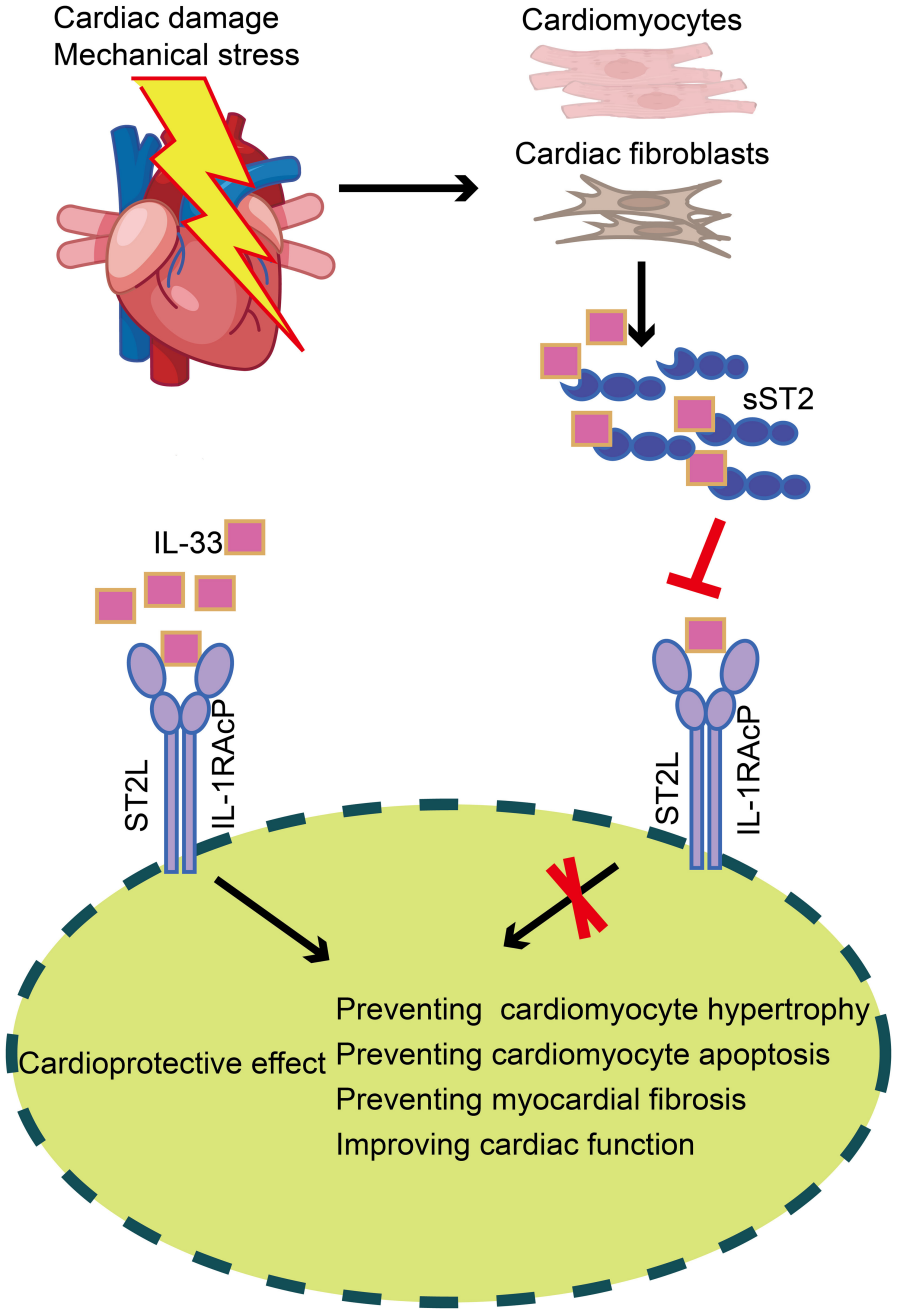
Production and role of sST2 in cardiac tissue. In cardiac tissue, IL-33 binds to a receptor complex composed of ST2L and IL-1RAcP, preventing cardiomyocyte hypertrophy, apoptosis, and myocardial fibrosis, thus improving cardiac function. On the other hand, cardiomyocytes and cardiac fibroblasts secrete sST2 when the heart is subjected to damage or mechanical stress. SST2 may bind free IL-33, substantially reducing the amount of IL-33 accessible for ST2L binding, thereby attenuating the cardioprotective effect of IL-33. sST2, soluble suppression of tumorigenicity; IL-33, Interleukin-33; ST2L, transmembrane isoform of suppression of tumorigenicity 2; IL-1RAcP, IL-1 receptor accessory protein.

ST2 is a member of the IL-1 receptor superfamily and exists in two different forms: a transmembrane receptor (ST2L) and a soluble receptor (sST2) (39). Interleukin-33, a cardiac fibroblast protein released by stromal cells in cardiac and extracardiac tissues, is the ligand of ST2. IL-33 binds to a receptor complex composed of ST2L and IL-1 receptor accessory protein, which prevents cardiomyocyte hypertrophy, apoptosis, and myocardial fibrosis, thereby improving cardiac function. On the other hand, cardiomyocytes and cardiac fibroblasts secrete sST2 when the heart is subjected to damage or mechanical stress. SST2 may bind free IL-33, substantially reducing the amount of IL-33 accessible for ST2L binding, attenuating the cardioprotective effect of IL-33, and ultimately contributing to myocardial fibrosis (Figure 4) (40). Serum sST2 is unaffected by potential confounding variables, including age, sex, body mass index, and comorbidities such as renal disease and diabetes (41), making it a promising biomarker. Relevant clinical investigations have confirmed that serum sST2 can be utilized as an additional parameter for the diagnosis and prognosis of cardiovascular illnesses such as coronary heart disease (42, 43), aortic dissection (44, 45), and HF (46, 47).

Among HFpEF patients, we found that serum sST2 exhibited a low diagnostic value compared to NT pro-BNP. However, strong evidence shows that sST2 provides a synergistic incremental value to NT-proBNP for the diagnosis of HF (48). Sinning et al. also revealed that combining biomarkers [(CRP+GDF-15+sST2)/NT-proBNP] could effectively distinguish HFpEF from HFrEF (17). Therefore, the combination of serum sST2 with other biomarkers may provide incremental value for diagnosing HFpEF and deserve further investigation. More importantly, we found that increased serum sST2 was strongly associated with an increased risk of adverse endpoints. According to Manzano-Fernández et al., individuals with high levels of sST2 (>0.72 ng/ml) had a greater risk of death than those with low levels (0.33–0.71 ng/ml), with an HR of 4.18 vs. 2.63 (21). To exclude the effects of possible confounders, some studies adjusted for variables such as age, sex, race, and NYHA class, particularly Najjar et al. (18), Shah et al. (19), and Manzano-Fernández et al. (21) adjusted for NT-proBNP. We pooled the adjusted HR and found that log sST2 remained a significant independent predictor of all-cause death and the composite endpoint of all-cause death and HF hospitalization in HFpEF patients. Subsequently, further determination of the optimal cut-off value of sST2 is necessary to provide accurate prognostic information to the patient. However, consensus on a cut-off value predicting adverse outcomes is lacking due to differences in population characteristics, sST2 assay and detection methods. Manzano-Fernández et al. demonstrated that an sST2 cut-off of 0.35 ng/mL provided the best risk prediction for all-cause death (21), while Pan et al. confirmed that the optimal cut-off value was 0.332 ng/mL (16). Therefore, more large-scale multicenter studies need to be conducted to establish the optimum cut-off values. Moreover, sST2 can provide incremental value to other biomarkers or risk prediction models. According to Fries et al., sST2 and NT-proBNP work better in combination than separately to predict the composite endpoint of all-cause death and HF hospitalization in HFpEF patients (49). Gao et al. (20) found that sST2 combined with other biomarkers appreciably improved the value of the ASCEND-HF risk prediction model for predicting all-cause death. Finally, sST2 may be a promising therapeutic target for HFpEF and a helpful tool for monitoring treatment response because of its remarkable association with the pathological mechanisms of myocardial fibrosis and adverse outcomes. To date, the two studies available on the use of LCZ696 in HFpEF patients (LVEF ≥ 45%) yielded opposite results. The PARAMOUNT trial showed no LCZ696 treatment-related change in sST2 at 12 and 36 weeks of treatment (50). In contrast, the PARAGON-HF study indicated a 4% reduction in sST2 after 16 weeks of curing, and the changes in sST2 were linked to patient prognosis (51). Accordingly, whether sST2 is a therapeutic target for HFpEF and a monitoring tool for treatment response deserves further investigation.

## Strengths and limitations

The strengths of our studies are as follows: Firstly, according to the NOS score, all of the included studies were of high quality, making our research findings more accurate. Additionally, subgroup analysis was used to find the source when heterogeneity was more than 50%, helping to identify variables affecting outcomes. In order to determine if sST2 is independently associated with poor endpoints, unadjusted and multivariable-adjusted HRs and the corresponding 95% CIs are pooled. Nonetheless, as a pooled meta-analysis incorporating retrospective and observational studies, this study is constrained by the inherent drawbacks of combining investigations, such as heterogeneous patient populations, inconsistent clinical features, and differences in sST2 assay kits, measurement methods, and units. Moreover, in the diagnostic analysis, The number of included studies was limited, and previous studies applied inconsistent cut-off values of sST2, preventing the pooling of results as a meta-analysis. Similarly, very few studies were included in the prognostic analysis, with only six being multicenter studies and the remaining being single-center, and none had a sample size of more than 400 cases. Also, the length of the follow-up period and adjustment variables differed considerably among studies. More importantly, the publication bias assessment confirmed the existence of a significant publication bias, limiting the ability to draw substantial conclusions. Therefore, prospective and multicenter studies with large-sample and extended follow-up periods are required to validate our results.

## Conclusion

In conclusion, serum sST2 exhibited poor diagnostic values in independently identifying HFpEF from healthy controls, hypertensive patients, and HFrEF patients. Nevertheless, it may provide incremental value to other biomarkers for diagnosing HFpEF and deserves further investigation. In addition, higher sST2 was strongly linked to an increased risk of adverse outcomes in HFpEF patients. In particular, log sST2 independently predicted all-cause death and the composite endpoint of all-cause death and HF hospitalization. Finally, and perhaps most importantly, the optimal threshold levels of serum sST2 need to be further determined to provide more accurate prognostic information.

## Data availability statement

The original contributions presented in the study are included in the article/Supplementary material, further inquiries can be directed to the corresponding author/s.

## Author contributions

GD and JL designed this meta-analysis. GD reviewed the manuscript. YS performed the meta-analyses and wrote the manuscript. YS and CL developed the search strategy and performed literature searches and screening. YS and XS was conducted the data extraction. CY and WQ evaluated the quality of the enrolled studies. All authors contributed to the article and approved the submitted version.

## Funding

This study was supported by grants from the National Natural Science Foundation of China (8207153216) and the Major Innovation Project of the China Academy of Traditional Chinese Medicine (CI2021A00903).

## Conflict of interest

The authors declare that the research was conducted in the absence of any commercial or financial relationships that could be construed as a potential conflict of interest.

## Publisher's note

All claims expressed in this article are solely those of the authors and do not necessarily represent those of their affiliated organizations, or those of the publisher, the editors and the reviewers. Any product that may be evaluated in this article, or claim that may be made by its manufacturer, is not guaranteed or endorsed by the publisher.
