# Supplementary material for: Diagnostic and prognostic value of serum soluble suppression of tumorigenicity-2 in heart failure with preserved ejection fraction: A systematic review and meta-analysis
Source: Front Cardiovasc Med. 2022 Sep 20;9:937291. doi: 10.3389/fcvm.2022.937291 (PMC9530661; doi:10.3389/fcvm.2022.937291)
Supplement: Supplementary file 1 [file Data_Sheet_1.docx]

**Supplemental materials**

1. **Supplementary Table 1: NOS Score of the Included Studies**

| **Study** | **Quality indicators from the NOS** | | | | | | | | **Total scores** |
| --- | --- | --- | --- | --- | --- | --- | --- | --- | --- |
|  | **Selection** | | | | **Comparability** | **Outcome** | | |  |
|  | **(i)** | **(ii)** | **(iii)** | **(iv)** | **(v)** | **(vi)** | **(vii)** | **(viii)** |  |
| Cui2018 | ☆ | ☆ | ☆ | ☆ | ☆☆ | ☆ | ☆ |  | 8 |
| Santhanakrishnan2012 | ☆ | ☆ | ☆ | ☆ | ☆☆ | ☆ | ☆ |  | 8 |
| Wang2013 | ☆ | ☆ | ☆ | ☆ | ☆ | ☆ | ☆ |  | 7 |
| Pan2020 | ☆ | ☆ | ☆ | ☆ | ☆ | ☆ | ☆ |  | 7 |
| Sinning2016 | ☆ | ☆ | ☆ | ☆ | ☆☆ | ☆ | ☆ |  | 8 |
| Najjar2019 | ☆ | ☆ | ☆ | ☆ | ☆ | ☆ | ☆ |  | 7 |
| Shah2011 | ☆ | ☆ | ☆ | ☆ | ☆ | ☆ | ☆ | ☆ | 8 |
| Gao2021 | ☆ | ☆ | ☆ | ☆ | ☆☆ | ☆ | ☆ |  | 8 |
| Manzano-Fernández2011 | ☆ | ☆ | ☆ | ☆ | ☆ | ☆ |  | ☆ | 7 |
| Sugano2019 | ☆ | ☆ | ☆ | ☆ | ☆ | ☆ |  |  | 6 |
| Roy2020 | ☆ | ☆ | ☆ | ☆ | ☆ | ☆ |  | ☆ | 7 |
| Song2008 | ☆ | ☆ | ☆ | ☆ | ☆ | ☆ |  |  | 6 |
| Sanders-van Wijk2015 | ☆ | ☆ | ☆ | ☆ | ☆☆ | ☆ | ☆ | ☆ | 9 |
| Chirinos2020 | ☆ | ☆ | ☆ | ☆ | ☆ | ☆ | ☆ | ☆ | 8 |
| Kanagala2020 | ☆ | ☆ | ☆ | ☆ | ☆ | ☆ | ☆ | ☆ | 8 |
| Moliner2018 | ☆ | ☆ | ☆ | ☆ | ☆☆ |  | ☆ | ☆ | 8 |

**For the** **study for diagnostic analysis, the NOS evaluation included:** (i) adequate definition of cases, (ii) representativeness of the cases, (iii) selection of controls, (iv) definition of controls, (v) comparability of **cases** and controls based on the design or analysis, (vi) ascertainment of exposure, (vii) same method of ascertainment for cases and controls, and (viii) nonresponse rate.

**For the study for prognostic analysis, the NOS evaluation included:** (i) representativeness of the exposed cohort, (ii) selection of the non-exposed cohort, (iii) ascertainment of exposure, (iv) demonstration that the outcome of interest was not present at the start of the study, (v) comparability of cohorts based on study design or analysis, (vi) assessment of outcomes; (vii) was follow-up long enough for outcomes to occur, and (viii) adequacy of follow-up of cohorts.

1. **Supplementary Table 2: Subgroup Analyses of All-cause Death Risk in the Per Unit Increase Group**

| **Subgroup** | **Grouping Status** | **Number of studies** | **Meta-analysis** | | | **Heterogeneity** | | **P-value of**  **difference between subgroups** |
| --- | --- | --- | --- | --- | --- | --- | --- | --- |
|  |  |  | **Effect size** | **95%CI** | **P-value** | **I^2^** | **P-value** |  |
| Ethnicity | Asian | 2 | 1.27 | 0.75-2.14 | p=0.374 | 79.8% | p=0.026 | 0.332 |
|  | Western | 2 | 4.79 | 0.34-66.53 | p= 0.244 | 92.6% | p=0.000 |  |
| Sex | Males  >50% | 2 | 4.16 | 0.22-77.36 | p=0.339 | 94.1% | p=0.000 | 0.473 |
|  | Males  <50% | 2 | 1.43 | 1.18-1.72 | p=0.000 | 0.0% | p=0.348 |  |
| Study design | Single  -center | 2 | 5.42 | 0.50-58.97 | p=0.165 | 90.2% | p=0.001 | 0.208 |
|  | Multicenter | 2 | 1.16 | 0.87-1.55 | p=0.310 | 87.1% | p=0.005 |  |
| sST2  detection method | ELISA | 3 | 1.52 | 0.94-2.45 | p=0.085 | 91.9% | p=0.000 | 0.671 |
|  | multiplexed assay | 1 | 1.76 | 1.9-2.85 | p=0.021 | — | — |  |
| sST2 unit | ng/ml | 3 | 2.49 | 1.11-5.59 | p=0.027 | 85.7% | p=0.001 | 0.030 |
|  | pg/ml | 1 | 1.02 | 1.01-1.03 | p=0.000 | — | — |  |
| Follow-up time | times>24 months | 1 | 20.24 | 4.88-83.99 | p=0.000 | — | — | 0.000 |
|  | times<24 months | 3 | 1.27 | 0.95-1.70 | p=0.114 | 84.3% | p=0.002 |  |

1. **Supplementary Figure 1: Publication Bias and Sensitivity Analysis of Risk analysis for** **All-cause Death**

**
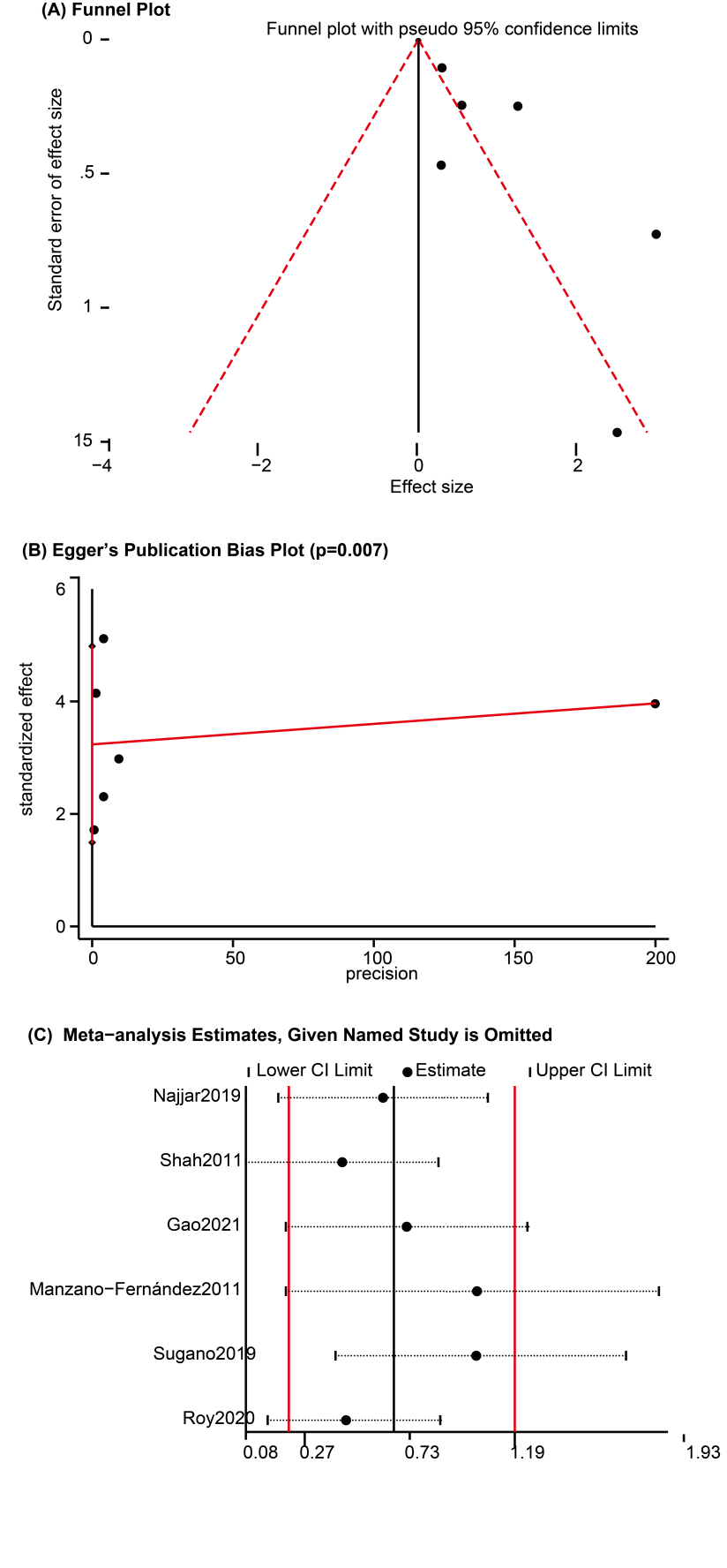
**

**Supplementary Figure 1A**: The Funnel plot was asymmetrical; **Supplementary Figure 1B**: the p-value of Egger's test was 0.007, indicating notable publication bias.

**Supplementary Figure 1C:** The sensitivity analyses indicated that none of the individual trials substantially impacted the pooled estimates.

1. **Supplementary Figure 2: Publication Bias and Sensitivity analysis of Risk Analysis for the Composite Endpoint of All-cause Death and HF Hospitalization.**

**
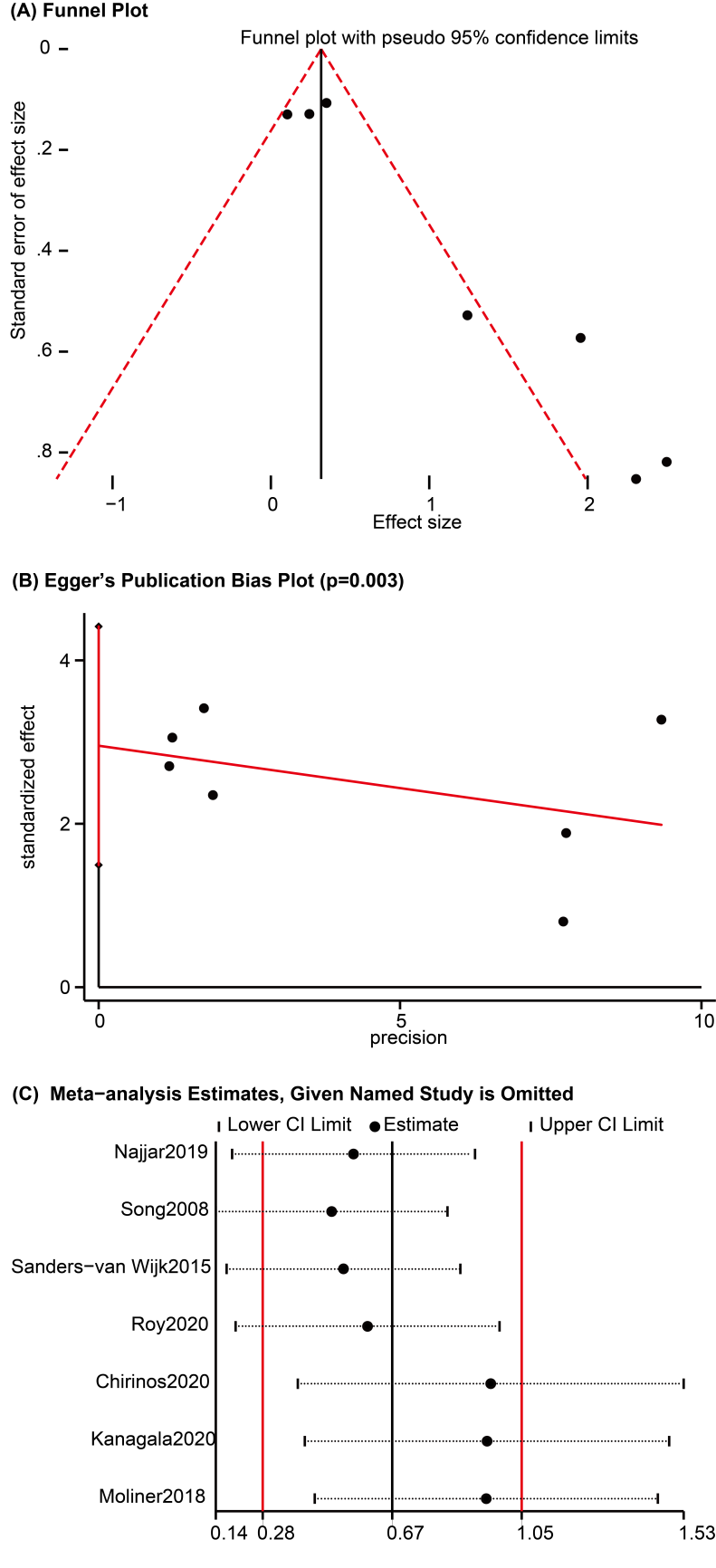
**

**Supplementary Figure 2A**: The Funnel plot was asymmetrical; **Supplementary Figure 2B**: the p-value of Egger's test was 0.003, indicating notable publication bias.

**Supplementary Figure 2C:** The sensitivity analyses indicated that none of the individual trials substantially impacted the pooled estimates.
